# Re-imagining the control of malaria in tropical Africa during the early years of the World Health Organization

**DOI:** 10.1186/s12936-015-0700-9

**Published:** 2015-04-24

**Authors:** Socrates Litsios

**Affiliations:** World Health Organization, Geneva, Switzerland

**Keywords:** Malaria, Africa, History, World Health Organization, Primary health care

## Abstract

This paper grew out of a meeting organized in September 2014 in London on ‘Re-imagining malaria’. The focus of that meeting was on malaria today; only afterwards did the idea emerge that re-imagining the past might serve as a useful way for guiding present re-thinking. Sub-Saharan Africa is the logical place for such a re-examination for, as argued in this paper, the approaches that emerged following the collapse of the global eradication campaign were available to WHO in the 1950s, but these were not pursued as Africa was not encouraged to seek solutions outside those being advocated for eradication elsewhere.

## Background

When the World Health Organization (WHO) was in the process of being established, its governing bodies were mostly preoccupied with problems being faced by countries that had been severely touched by World War II. Italy was still suffering high rates of malaria due to retreating German soldiers having destroyed the system of drainage in the Pontine Marshes; Egypt faced a cholera outbreak that demanded urgent attention; post-war Eastern Europe was witnessing dramatic increase in deaths due to tuberculosis due to miserable living conditions that the war had created. In this context malaria emerged as a priority, not only due to the high morbidity and mortality that it caused around the world, but because new tools (especially that of DDT) gave promise that it could be controlled without an undue financial burden.

Tropical Africa was not touched by the war to the extent that Europe and Asia had been. Furthermore, being composed mostly of colonial states, it lacked the political weight that the other regions of the world possessed. Not surprisingly, there were delays in developing programmes there and when efforts finally were initiated they were not as heavily invested as in other areas of the world. While malaria was clearly a major problem in tropical African countries, by the time it was addressed there progress in other parts of the world had been so promising as to convince some that equally encouraging results could be obtained there as well. As is well known, this did not happen. What did happen, instead, malaria control in this area of the world became “an aborted campaign for eradication” [1].

When malaria in tropical Africa was finally addressed in more realistic terms, which started in the 1970s, many of the principles and approaches then advocated were similar to those that had been advocated before the war in Asia and Africa. It is this similarity that leads us to ‘imagine’ a different start for Africa.

In no way will this paper deal with the technicalities of malaria control, e.g. the choice of insecticides and drugs, but instead it will focus on the fact that there was in place sufficient awareness of alternative approaches that could have been explored.

The paper is structured chronologically; it begins with an account of the pre-war era before going on to the early years of WHO that preceded the launching of the global eradication campaign. This is followed by a discussion of the post-eradication era. The paper concludes with an imaginary history.

### Pre-World War II developments of note

While malaria was certainly one of the big killers in tropical Africa, more attention was paid by the colonial governments to sleeping sickness owing to its greater economic importance. As portrayed by Julian Huxley after his visit to Africa in 1929:

*Now the malaria mosquito is bad enough; but malaria does not drive cultivation out of a country like the fly-disease of cattle, nor does it kill wholesale like the tsetse of human sleeping sickness. And finally it is a more orderly and controllable creature* [2]*.*

The League of Nations Health Organization (LNHO) began its study of sleeping sickness in the early 1920s. Although the LNHO established a Malaria Commission soon thereafter, its first priority was Europe; only in the early 1930s did Ludwik Rajchman, Director of the LNHO, move to “integrate Africa into LNHO work…” [3]. This was facilitated by the Government of South Africa proposing that a conference similar to the rural health one held in Europe be organized for the countries and territories of virtually all of Africa. It took place in Cape Town in November 1932. A follow-up conference took place three years later, in November 1935. Like the 1932 meeting, it was attended by both African colonial officials and representatives of international organizations, including the League’s Health Section and the Rockefeller Foundation. The 1935 conference stressed the need for a broad based approach to health, which rested on economic advancement. Concerning malaria, it noted that additional research on African malaria was needed but “…*it must not be forgotten that, without raising the economic status of the vast bulk of the population of Africa as a whole, there can be no hope of applying successfully on a continental scale the results of research or of markedly improving the position of great populations with regard to malaria as a disease*” [4].

While another conference was not organized in Africa, mention must be made of a LNHO-sponsored conference that took place in Bandoeng, Indonesia in 1937 on the subject of rural hygiene, especially as Bandoeng’s discussions concerning malaria offered useful advice that was relevant to some situations in Africa and thus appropriate for inclusion here. The Technical Committee on Malaria at Bandoeng was chaired by Paul Russell, Rockefeller Foundation staff member; also present was Emilio Pampana, who, on joining WHO in 1948 took charge of its malaria programme. The Committee placed emphasis on indicating “some practical lines of procedure as regards public policy in dealing with malaria” in rural populations. Given the variability across the countries of the Far East no attempt was made to standardize anti-malaria procedure as this would be “highly inadvisable”. Each country “*must determine for itself, with the help of personnel specially trained in malariology, the most logical plan of campaign within its own borders, having due regard for general principles, for the funds and staff available, the invariably focal distribution of the disease, and the opportunities for enlisting local cooperative assistance*” [5]. Nevertheless, the first responsibility of the Government in any malaria campaign is to “*save from death and relieve from physical distress the malarious sick by making anti-malarial treatment readily available*”. During epidemics “free treatment” should be made available for all [5].

Furthermore, the responsibility for malaria control should rest “*squarely on the minister or other officer in charge of the public health policy of a country and not on the technical exper*t”. Practical demonstrations of malaria control should be used as a means of arousing the interest of the lay (and even health) administrators. Where malaria control programmes are poorly developed, “sufficient funds [should] be allocated for at least five years to carry out a model project” [5].

In addition to extending the free distribution of cinchona products, every effort should be made “*to enlist the aid of the people themselves in minor control methods, and to explore cheaper methods of control which use time more than money. Persistence rather than perfection in control is required*” [5].

Farmers could play a major role if taught “*such minor naturalistic methods as herbage packing with green-cut vegetation; fostering natural enemies such as fish; removing sheltering vegetation; shading breeding-places by cultivating certain plants or using coconut palm leaves, or woven bamboo mats, where the dangerous mosquito requires sunlight; periodical sluicing of small streams to eliminate mosquito breeding*” [5].

A number of recommendations were made at Bandoeng concerning the need for further research:

1. While the systematic classification of anophelines is on a sound basis, there is a serious lack of information as to the bionomics even of those species known to be dangerous malaria carriers. Success in developing cheaper methods of naturalistic control must depend on a much fuller knowledge of the habits and intimate habitats of anophelines. This applies not only to antilarval measures, but also to such potentially useful methods of attacking adult mosquitoes as insecticidal spraying.
2. A much more definite understanding of the relationship between malaria, malnutrition, famine and poverty is required, as well as further elucidation of the factors concerned in malarial immunity especially in so-called racial immunity.
3. A good deal more investigation is required to develop practical mosquito-nets for rural areas in the tropics, to forecast epidemics, and to make the use of zooprophylaxis practicable [5].

In another context Russell expressed his belief that “*without sufficient health-knowledge among laymen, personal and community hygiene is impossible, necessary appropriations for public health measures are not voted, salutary laws are not made or obeyed, sanitary progress is delayed*” [6]. This was consistent with the project that he initiated in the early 1930s in the Philippines, where, feeling “*strongly that control work must be locally desired and locally carried out*”, he wished to engage community leaders in the development of local control schemes [7]. Although this project lasted for no more than one or two years, it did lead Russell to develop *A Malaria Primer* to be used by students and teachers. This primer was in “the nature of a profusely illustrated elementary handbook” [8].

Also of relevance are the early efforts in which quinine was used to treat Africans. Following the ‘massive usage’ of quinine that brought to an end an epidemic in Ruanda-Urundi in 1934–1935, there was “a broader acceptance of mass chemical treatment in Africans in epidemic conditions” [1]. The use of quinine for curative purposes was extended to cover non-epidemic situations as was the case in the Gold Coast, where the British offered quinine “at a cost price through the Post Offices” [1]. This programme continued into the Second World War; a similar programme of distribution developed in Nigeria.

African populations also had recourse to a “*wide array of plant-based medicines, some of which had the ability to reduce parasite loads or alleviate malaria symptoms. These medicines did not clear infections, yet many were palliative*” [1]. Also, tropical Africans “*had devised ingenious methods to reduce the annoyance of buzzing and biting mosquitoes, particularly during evenings and nights when the insects prevented sound sleep*” [1]. These methods included the setting of a smoky fire, the use of plants for fumigation and mosquito nets fashioned from cotton cloth and raffia palm fronds.

Marshall Barber, in his *A Malariologist in Many Lands*, published in 1946, provides a kaleidoscopic picture of malaria as he saw it in his long career with the Rockefeller Foundation. As described by Russell – Doctor Barber has been chasing “malaria plasmodia” and their anopheles vectors for half a century, prying into their secret habits and paving the way for their destruction. He has outwalked more mosquito-collectors in more countries than any other malariologist of record [9]. Barber’s concluding pages are striking as they are dedicated to the belief that malaria can be conquered through education. Characterizing the “mosquito enemy” as a “fragile thing, vulnerable on land and in water and beset with myriads of natural enemies”, it is “human ignorance” that allows this enemy to survive. His experience persuaded him “*that if only one could convince people that mosquitoes carry malaria and teach them a few simple means of protection, a vast proportion of the disease would disappear, almost overnight*” [9]. He did not recommend education as a complete substitute for anti-malaria measures conducted by the state or other health institutions; education was to “*enable people to undertake some part of the work themselves for they may have to wait for generations for the State to get around to them, especially in communities impoverished by famine or war*”. Barber wrote that “*we should not be discouraged if it requires a generation or two among some peoples to accomplish much education … a good way to begin is to utilize the natural curiosity of children*”. He proposed that such education begin by the use of simple means – “like a fruit-jar, or other aquarium, set in the schoolroom window, where pupils could see the various stages of mosquito development – elementary instruction which would prepare their minds for learning the rudiments of malaria transmission” [9].

Barber’s book was published in 1946, the year that WHO’s programme began to take shape. Perhaps as a sign of things to come, Russell, who wrote the foreword for Barber’s book, made no reference to Barber’s conclusions but simply described his book as one that gave a “*simple account of the author’s experiences in his malaria work in various parts of the world in order to promote the ‘unlearning’ of some misconceptions and the learning of some simple truths about this important disease*” [9].

### Pre-eradication – WHO’s first years

Malaria was identified by the Interim Commission (IC) that was established to set out the early programme of WHO as one of the organization’s top priorities. By this time, Pampana had become head of the WHO unit responsible for malaria while Russell was still with the Rockefeller Foundation. The two worked together to help guide the agenda of work of the WHO Expert Committee that was established by the IC, as well as to determine its membership.

Brock Chisholm, WHO’s first Director General, in his annual report for 1948, wrote:

*By means of advice furnished by the Expert Committee on Malaria, field services and visits of individual experts, the Organization has assisted governments in carrying out malaria control. It has thus paved the way for action to be taken by the United Nations through its Economic Commissions, and had caused the world to think, perhaps for the first time, in terms of worldwide eradication of malaria* [10].

Van Zile Hyde, who earlier had been Chief, Health Division, UNRRA, Director of the Point IV Health Program within the US State Department, and was then Chief, Division of International Health, USPHS, and an ardent supporter of malaria being a top priority in WHO’s programme, informed Chisholm that in his opinion, “*a total world malaria eradication programme can be developed and pushed to conclusion*” [11].

Within a few years, Chisholm attempted to bring together malaria along with the other top priorities under the umbrella of integrated rural health services, whose focus was on the rural health unit, which was defined as an organization providing or making accessible, under the direct supervision of at least one physician, the basic health services for a community, which included maternal child health, communicable disease control, environmental sanitation, maintenance of records for statistical purposes, health education of the public, public-health nursing, and medical care (to an extent varying with the needs of the area and the accessibility of larger hospital centres).

In 1953, when the environmental sanitation expert committee was called upon to study the sanitation problems of small communities “in under-developed countries and methods of solving these problems”, Marcolino Candau, recently elected Director-General of WHO, noted in his introductory remarks that experience had pointed “to the inevitable conclusion that a programme of rural sanitation cannot be successful without the active participation of the local community. It is necessary for all health workers at every level to participate in well-designed programmes of health education of the rural population” [12].

The committee, which was chaired by George Macdonald (whose mathematical models supported the belief that malaria could be eradicated), believed that it “*should be axiomatic to integrate environmental sanitation programmes in underdeveloped areas with general community development, with particular reference to agriculture*”. It recognized that the first step in any programme “should be the elimination of those factors which are the most important agents in the transmission of diseases” [12]. It recommended that the first basic steps towards the provision of a safe environment in rural areas and small communities should be action to:

- Provide adequate supplies of safe drinking-water;
- Provide for the safe disposal of human excreta; and
- Control the insect and animal vectors of disease where they are of significant importance. This activity should not take such precedence in programmes as to exclude action in the safe disposal of excreta and in the provision of safe water supplies.

Leaders in tropical medicine were also seeing the need to go beyond contributing to the advancement of disease control methods in individual countries and local communities. The “outstanding service of tropical medicine to mankind … is in international health. The geographic areas of greatest interest to tropical medicine are the underdeveloped regions. These are just the places where shortcomings in economics, agriculture, education and health keep countries at the sort of disadvantage that breeds discontent and leads to conflict. There is now a belated realization that great differences in status between countries in these respects are a world hazard” [13].

At the same time that the organization was highlighting the importance of an integrated approach to rural health, a conference was organized in Kampala, Uganda in 1950 to explore options for malaria control in tropical Africa. The historical importance of this meeting cannot be over-exaggerated. Pushing aside all of the worries that disturbing the immune status of indigenous populations might cause more harm than good, the Conference recommended that “*malaria should be controlled by modern methods, as soon as feasible, whatever the original degree of endemicity and without awaiting the outcome of further experiments*”, while pointing out “that the higher the degree of endemicity, the more important it is to establish a malaria control organization so that there may be continuation of the work until the progress of control might allow relaxation without danger of an outbreak of malaria” [14]. The only aspect of integration that was briefly discussed was the link between malaria control and agricultural development.

A Global Campaign for the eradication of malaria was soon conceived; its supporters justified the heavy investment in such a campaign on the grounds that “eradication represented a large up-front capital investment that would be quickly returned by the elimination of long-term recurrent expenses associated with maintaining control measures indefinitely” [15]. Given the expectation that eradication would be a time-limited affair, absolutely no attention was paid to other programmes, health or otherwise. Although the official WHO line is that tropical Africa was never included in that campaign, the words of Francisco Cambournac, Regional Director for Africa, belie that interpretation. Reporting to the 19^th^ session of the WHO Executive Board in January 1957, he referred to the “*temporary exclusion of the African Region from WHO’s world-wide malaria eradication plans*” [16].

### Post-eradication malaria control

The global eradication campaign came to an end for all intents and purposes in 1969. While there remained countries that still hoped for eradication in the short-term, attention was now given to countries where “time-limited eradication is impracticable at present”; an interregional conference held in Brazzaville in 1972 on this subject represented the first serious post-eradication era to address malaria control in tropical Africa. The meeting judged it “probable” that the association of chemotherapy with residual spraying would be the “only way of gradually eliminating malaria from those tropical areas where the degree of transmission is extremely high”, adding, however, that it was “not possible to predict the success or failure of this combined method on the basis of existing knowledge and practical experience” [17]. Revealingly, it was noted that the “knowledge of the technical feasibility of antimalaria programmes” had “changed little since the beginning of large-scale campaigns with a view to eradication” [17].

The basic health services were seen to have a role to play in treating malaria cases, in mass drug administration, collecting data required for evaluation, and health education. Health education was seen as the key for realizing the “great potential” that exists in rural communities for “self-help in improving their health conditions when well-motivated …” [17]. Health education “must take into account all the existing cultural attitudes, beliefs, and behaviour characteristics related to life and work”. Recognizing that the “values held, and the judgement criteria used, by populations – factors which may be responsible for determining health actions and beliefs – sometimes differ from those of malaria control personnel”, it was necessary “to consider with care what approaches should be used to ensure the desired cooperation and participation of the people” [17]. The conference recommended that “*pilot studies be promoted on motivating rural communities to undertake self-help activities on malaria control*” [17].

With the adoption of the primary health care (PHC) approach even greater attention was given to health education and the role that communities could play in malaria control. American malariologists took the lead in exploring the implications of the PHC approach for malaria control in tropical Africa. Several years before WHO organized a study group on this subject (see below), America “*had decided to integrate all future anti-malarial measures into the PHC system*” [1]. At the same time it was recognized that any “proposal to encourage African communities to take on a programme of self-help that included screening, bed nets, pyrethrum coils and sprays, and anti-malarial drugs would have to negotiate in the grand diversity of African rural societies. What seemed possible in the compact villages of Northern Nigeria, in which village chiefs and emirs exercised hierarchical control, seemed impossible in the dispersed individual compounds of Kenya” [1].

USAID developed a manual in 1982 on malaria control in primary health care in Africa and organized a workshop on the subject in June 1983. The manual suggested that educational activities be used to stimulate appreciation of early treatment, motivate compliance with chemotherapeutic regimens, and, when appropriate, motivate villagers to adopt new patterns of behaviour that might reduce the likelihood of infections at a later time. In the final analysis, however, “the ultimate success of the programme will depend less upon the message from outside the community than upon the extent to which community leaders and organizations are able to develop support for the programme and understanding of its details on the part of the community residents” [18].

The workshop further explored the implications of the PHC approach for malaria control [19]. It endorsed the idea that AID support national training of PHC and malaria workers in concepts and methods for developing and implementing educational approaches. Such approaches should emphasize preventive and protective actions that individuals and communities could take for malaria control, such as reduction of sources for vector breeding, physical personal protection techniques (screens, bed nets, repellents), effective use of drugs and early notification to and contact with primary health workers and malaria workers for treatment.

Later in 1983, a WHO Study Group on Malaria Control as part of Primary Health Care considered the planning of malaria control in that context and, particularly, the collective experience of countries, in order to identify practical approaches that could be applied at various levels and those which needed to be further developed [20]. The group explored the same issues that USAID had been investigating, in particular the role of the community in anti-malarial activities, which it saw as requiring the promotion of the general awareness of the community of the importance of prompt and effective treatment of malaria cases, for which community health workers needed to possess the necessary diagnostic skills and technical and supervisory support. Individuals in the community should be motivated to use mosquito nets and repellents as safeguards against malaria. Countries were urged to support efforts to create community awareness and involvement in activities affecting the quality of life.

Aware of the fact that in many areas of Africa private channels of distribution of anti-malarial drugs reached the periphery more effectively than did the health services, the WHO Malaria Expert Committee that met in 1985 recommended that the health service concentrate its efforts on providing adequate information about the use of drugs, improving accessibility to appropriate treatment, and eventually regulating and controlling the drug trade [21]. The next Expert Committee meeting (the 19^th^ session), which took place in 1989, stressed the point that communities could not solve the malaria problem on their own through self-protection and community prevention activities, as there is a need for timely diagnostic and therapeutic services, including a referral system capable of managing treatment failures and severe and complicated malaria cases, functions which only a well-established health system can provide.

The importance given to diagnostic services and treatment facilities led the committee to recommend that, since self-treatment is practiced in most communities, “*all possible channels should be used to teach the proper use of drugs, including community leaders, traditional birth attendants, pharmacists or drug vendors, school teachers and radio broadcasters, as well as health professionals (including private practitioners*)” [22]. American experience in Africa had led their malariologists to conclude that “parents and communities must be involved in the process of disease recognition, therapy, and prevention” if the existing patterns of childhood morbidity and mortality were to be improved [23].

The early 1990s witnessed increased attention to African malaria. Of particular relevance, given its broad scope, was a study organized by the American Association for the Advancement of Science Sub-Saharan African Program under a cooperative agreement with USAID entitled *Malaria and Development in Africa: A Cross-Sectoral Approach*. The final report consists of some 40 pages of recommendations supported by 20 background papers! The attention given to ‘human capital’ and ‘community participation’ dominates the report. Human capital was seen as “the most important investment to be made for malaria control”, for which the “participation of communities” was judged to be “critical to programme success and sustainability” [24].

Several pages of recommendations are devoted to the importance of “recognizing the social and environmental factors that influence behaviour” as part of the community-based approach needed to sustain malaria control. Arguing that a “top-down approach alone will not work”, a “community-based strategy must be employed recognizing that individuals are already making decisions for themselves and their families regarding malaria prevention and control, and only programmes that are consistent with their interest will ultimately be sustainable” [24].

### Imagining a different start for Tropical Africa

What is clear from the above is that many of the approaches of the 1970s were already advocated in the 1930s, in particular those related to popular health education and local application of anti-mosquito and personal protection measures. It is due to this that we can imagine that nothing in principle prevented their application two decades earlier, i.e. in the 1950s.

The imaginary start begins with several of Russell’s observations. First, that there were “more difficulties in Africa than elsewhere, since no large area had yet been cleared by the methods advocated by WHO and it was therefore impossible to plan for country-wide eradication with any assurance” [25], and most importantly, as already cited, his strong earlier (1937) belief that control work must be locally desired and locally carried out. A region-wide strategy would certainly have been put in place but what exact shape it would have taken is not certain. A first step would have been to develop a more refined map of the different endemicity levels throughout tropical Africa, one linked with basic ecological determinants such as climate, land cover and use, and population-related factors. Intensive studies of *Anopheles gambiae* would be associated with such mapping, especially in light of early evidence suggesting that *gambiae* was a complex made up of several species [26]. Two natural partners in such studies would have been the Commission for Technical Cooperation in Africa South of the Sahara (CCTA, as derived from its French title, Commission de Coopération Technique pour l’Afrique), which had already co-sponsored the 1950 Kampala malaria meeting, and the Scientific Council for Africa South of the Sahara (CSA as derived from its French title, Conseil Scientifique pour l’Afrique).

The CSA was concerned with promoting scientific activity in Africa by “encouraging more long-term research on key problems of which the solution might allow economic and social development to proceed more rapidly and with fewer false steps” [27]. It also sought to put the scientific centres and individuals in closer touch with each other. Together, the two organizations arranged more than 40 major meetings concerning a wide variety of subjects including maps and surveys, geology, meteorology, water and soils, medical cooperation and education, food and nutrition, rural welfare, statistics, housing, and labour. Some of the inter-African conferences resulted in the setting up of regular systems for consultation and joint action, as was the case for tsetse and trypanosomiasis. The two organizations also expressed interest at the 17^th^ session of the Executive Board in January 1956 in establishing an “inter-African scientific consultative committee for health to cover also social and cultural aspects” [28].

Continuing with the imaginary trip, the recognition that control work must be locally carried out would have led to attention being given to self-help. Given the diversity of peoples across Africa, it is clear that the participation of social/cultural anthropologists and ethnologists would have been given top priority. In fact, as noted in Lord Hailey’s report *An African Survey* published in 1945 – “In Africa there has developed a general recognition that policies which do not take into account the nature of the native societies to which they are applied are apt to provoke unforeseen and un-welcome reactions” [29]. Social/cultural anthropologists were seen as having particular relevance in indicating “*how a desirable reform may be brought about in such a way as to harmonize with the custom of the people whom it affects*” [29]. In order to improve the relevance of anthropological work to the practical needs of governments, the “bodies in Europe and America, which promote anthropological work” were called upon to better coordinate official and private inquiries” [29]. The subjects judged to be of particular importance at that time were malnutrition, population and soil erosion.

Lord Hailey identified a number of teaching and research institutions engaged in social anthropological studies in Africa. These included the Royal Anthropological Institute in London, the University of Manchester, several universities in South Africa, Northwestern and Yale University, Sorbonne’s Institut d’Ethnologie, and the State Universities of Ghent and Liege. Several of these had received funding earlier from the Rockefeller Foundation with a particular focus on Africa. In this imagined past, the Foundation would have extended its interest in malaria by encouraging these institutions to contribute to malaria control efforts in Africa. That the African Region of WHO was sympathetic to this development is suggested by what Francois Daubenton, its first Regional Director, had to say to the Executive Board in January 1954: it was impossible to consider health and disease in Africa as isolated factors; the environment, sanitary engineering problems, and social and anthropological conditions had also to be taken into account [30]. Pierre Dorolle, Deputy Director General of WHO, went further; he wrote of the “*absolute necessity to associate ethnological studies with all health actions*” [31]. Dorolle was one of the very few individuals still engaged in international health work who had participated in the 1937 Bandoeng Conference.

Dorolle managed to engage Jean-Paul Lebeuf, a very eminent French ethnologist, to work for WHO’s African regional office for several years in the early 1950s. At the end of his stay with WHO, Lebeuf wrote *L’Application de l’Ethnologie à l’Assistance Sanitaire*, in which many references are made to Dorolle’s papers on the subject. In turn, Dorolle drew upon Lebeuf’s work to illustrate the role of ethnologists in public health work, especially concerning health education of the public.

There were other anthropological studies contributing directly and indirectly to knowledge useful for malaria control. One publication, Murdock’s *Africa: its people and their culture history*, was recommended by Mansell Prothero, a leading geographer, “as essential reading for those engaged on malaria work in Africa”, as it provided a “synthesis of existing knowledge of the social relationships, religious beliefs and economic activities of African peoples” [32]. Murdock was an American anthropologist who chaired Yale University’s Department of Anthropology from 1938 to 1960. Prothero himself studied land use, population distribution and population mobility in tropical Africa and the impact of such movements on disease transmission in Africa, particularly that of malaria. His promotion of Murdock’s studies was included in his paper on human ecology in Africa as it related to malaria control, a subject which in the last decades has again re-surfaced.

What is clear from the above is that anthropologists were available who were seriously interested in working on malaria control projects had they been given the chance to do so, but the global campaign to eradicate the disease as quickly as possible left no room for such studies to be carried out.

Returning to the question of self-help, a good starting point would have been the report prepared at the request of the LNHO on naturalistic measures in the control of malaria in order to identify those measures that might have application in the African setting [33]. Other measures would have been uncovered by field work as later was the case with the use of indigenous fungus to attack *Anopheles larvae*, as studied by the International Centre of Insect Physiology and Ecology in Kenya [34].

Past experiences with health education as applied to malaria control would also have been reviewed. One experience particularly relevant to Barber’s call for educating children was the work of Wilhelm Schüffner in Malaya. He attempted to control malaria by destroying adult anophelines, not by means of insecticides, but by catching them by hand inside human habitations. This responsibility was given to schoolchildren who “were taught to identify (Anopheles) *sundaicus*, *hyrcannus*, *annularis*, *aconitus* and *vagus*” [35].

What is perhaps least clear is the rationale or selecting which situations to address first and how malaria control would be related to other priorities. It was easy for Cambournac to stress the importance of extending malaria control campaigns “as much as possible, leaving behind no area uncovered”, but actually delivering on such a promise was clearly impossible [36]. Furthermore, as pointed out by Van Zile Hyde, who represented the United States on the Executive Board, malaria eradication “was not the panacea for all ills. A country might have other health problems of even higher priority, and WHO, in the circumstances, would be taking an unrealistic view if it continued to insist on the rigid application of the criteria for participation in the proposed eradication programme” [28]. He went on to suggest that WHO “should devote more attention to environmental sanitation so as to eliminate the source of the menace”, i.e. vector breeding sites [28].

Other priorities included yaws, tuberculosis, nutrition, maternal child health and public health administration. The participation of UNICEF was crucial for these as well as for the regional malaria programme. Any new approach to malaria control would have had to be coordinated with the UNICEF’s priorities as well as those of other UN system organizations, in particular that of the FAO.

Imagining cooperation among the colonial powers and the new governments as colonial Africa became independent is perhaps the greatest challenge in this expose, but given the universal concern with malaria, had the US invested in Africa as it did for malaria work elsewhere, such a leap of imagination becomes a little less fanciful. But such assistance would not have been enough. As Paul-Marc Henry (Director of the CSA) noted in 1962: the brutal fact is that without some permanent connection with external sources of finance and administrative “know-how” through existing bilateral channels, or other machinery yet to be created, vast areas of Africa run the risk of a crippling paralysis which no amount of technical assistance or welfare work can expect to counteract” [37]. In the last analysis, according to Henry, “the United Nations is the only organization through which the problem of the development of Africa can be discussed in a world-wide context…. The African governments have a right to ask for long-term measures which would guarantee that over a period of years their essential economic and technical dependence will be turned into a normal partnership on equal terms” [37]. In this imaginary past, the United Nations family would have taken up the challenge of tropical Africa more seriously than it did.

## Concluding comments

It is impossible to know what degree of success would have been achieved in this imaginary past, but one thing that is certain is that it would have replaced almost certain failure with a process during which the public would have learned about malaria and health workers would have gained invaluable experience. Perhaps even Barber’s prediction that a well-educated public could lead to a vast proportion of the disease disappearing would have been proven correct. Whatever would have been achieved would have served as a more solid base to build on than what actually was realized.

## References

[1] Webb J (2014). The Long Struggle Against Malaria in Tropical Africa.

[2] Huxley J (1931). Africa View.

[3] Borowy I (2009). Coming to Terms with World Health: The League of Nations Health Organization 1921–1946.

[4] League of Nations (1936). Report of the Pan-African Health Conference, Johannesburg 20 to 30th, 1935. Quarterly Bulletin of the Health Organization.

[5] League of Nations (1937). Report of the Intergovernmental Conference of Far-Eastern Countries on Rural Hygiene.

[6] Russell P (1941). Some social obstacles to malaria control. Indian Medical Gazette.

[7] Anderson W (2006). Colonial Pathologies: American Tropical Medicine, Race, and Hygiene in the Philippines.

[8] The Rockefeller Foundation. Annual Report 1932. New York (http://www.rockefellerfoundation.org/uploads/files/0cc6dac7-6d59-44f9-af7c-f92d42001772-1932.pdf).

[9] Barber M (1946). A Malariologist in Many Lands.

[10] WHO (1948). Annual Report of the Director-General to the World Health Assembly and to the United Nations Official Records of the World Health Organization No 16.

[11] Jackson J (1998). Cognition and the Global Malaria Eradication Programme. Parassitologia.

[12] WHO (1953). *Expert Committee on Environmental Sanitation*, Third Report.

[13] Sawyer W (1951). Medicine as a social instrument: tropical medicine. N Engl J Med.

[14] WHO (1950). Report of the Malaria Conference in Equatorial Africa, Kampala, Uganda, Geneva, World Health Organization (WHO/Mal/69).

[15] Packard R (2007). The Making of a Tropical Disease.

[16] WHO. Executive Board 19^th^ session, Minutes of the Fourth Meeting, EB/19/Min/4 Rev. 1.

[17] WHO (1974). *Malaria control in countries where time-limited eradication is impracticable at present.* Report of a WHO Interregional Conference.

[18] USAID (1982). Manual on Malaria Control in Primary Health Care in Africa.

[19] AID Malaria Strategy Workshop, June 7–10, 1983 Columbia, 107 pages. Prepared for Agency for International Development by Insect Control & Research, INC, Baltimore, Maryland, September 1983.

[20] WHO (1984). *Malaria control as part of primary health care.* Report of a WHO Study Group.

[21] WHO (1986). *Expert Committee on Malaria*, Eighteenth Report.

[22] WHO (1992). *Expert Committee on Malaria*, Nineteenth Report. WHO/CTD/92.1.

[23] Breman JG, Campbell CC (1988). Combating severe malaria in African children. Bull World Health Organ.

[24] Malaria and Development in Africa (1991). A Cross-Sectoral Approach.

[25] WHO (1955). Eighth World Health Assembly.

[26] Coetzee M, Craig M, Le Sueur D (2000). Distribution of African malaria mosquitoes belonging to the *Anopheles gambiae* complex. Parasitol Today.

[27] Worthington EB (1983). The Ecological Century: A Personal Appraisal.

[28] WHO. Executive Board 17^th^ session, Minutes of the Fourth Meeting, World Health Organization, Geneva, EB17/Min/3/ Rev.1.

[29] Hayley M. An African Survey. Oxford University Press, 1945

[30] WHO. Executive Board 13th session, Minutes of the Eighteenth Meeting. World Health Organization, Geneva, EB13/min/18 Rev.1.

[31] Dorolle P (1953). Ethnologies et problèmes sanitaires. Revue Internationale de la Croix-Rouge et Bulletin international des Sociétés de la Croix Rouge.

[32] Mansell Prothero R. Some Aspects of Human Ecology in Africa Relevant to the Planning of Malaria Eradication Programmes, WHO/Mal/315. 6 October 1961.

[33] Hackett LW, Russell PF, Scharff JW, Senior White R (1938). The present use of naturalistic measures in the control of malaria. Bulletin of the Health Organisation of the League of Nations.

[34] MacCormack CP (1984). Human ecology and behaviour in malaria control in tropical Africa. Bull World Health Organ.

[35] Litsios S (2014). The Tomorrow of Malaria.

[36] WHO. Executive Board 21^st^ session, Minutes of the Sixteenth Meeting, World Health Organization, Geneva, EB21/Min/16 Rev.1.

[37] Henry PM (1962). The United Nations and the problem of African development. Int Organ.

